# Comparisons of Non-Gaussian Statistical Models in DNA Methylation Analysis

**DOI:** 10.3390/ijms150610835

**Published:** 2014-06-16

**Authors:** Zhanyu Ma, Andrew E. Teschendorff, Hong Yu, Jalil Taghia, Jun Guo

**Affiliations:** 1 Pattern Recognition and Intelligent System Lab., Beijing University of Posts and Telecommunications, No. 10 Xitucheng Road, Beijing 100876, China; E-Mails: hongyu@bupt.edu.cn (H.Y.); guojun@bupt.edu.cn (J.G.); 2 Computational Systems Genomics, CAS-MPG Partner Institute for Computational Biology, Shanghai Institute for Biological Sciences, Chinese Academy of Sciences, 320 Yue Yang Road, Shanghai 200031, China; E-Mail: a.teschendorff@ucl.ac.uk; 3 Statistical Genomics Group, Paul O’Gorman Building, UCL Cancer Institute, University College London, 72 Huntley Street, London WC1E 6BT, UK; 4 Communication Theory Lab., KTH - Royal Institute of Technology, Osquldas väg 10, 10044 Stockholm, Sweden; E-Mail: taghia@kth.se

**Keywords:** non-Gaussian statistical models, dimension reduction, unsupervised learning, feature selection, DNA methylation analysis

## Abstract

As a key regulatory mechanism of gene expression, DNA methylation patterns are widely altered in many complex genetic diseases, including cancer. DNA methylation is naturally quantified by bounded support data; therefore, it is non-Gaussian distributed. In order to capture such properties, we introduce some non-Gaussian statistical models to perform dimension reduction on DNA methylation data. Afterwards, non-Gaussian statistical model-based unsupervised clustering strategies are applied to cluster the data. Comparisons and analysis of different dimension reduction strategies and unsupervised clustering methods are presented. Experimental results show that the non-Gaussian statistical model-based methods are superior to the conventional Gaussian distribution-based method. They are meaningful tools for DNA methylation analysis. Moreover, among several non-Gaussian methods, the one that captures the bounded nature of DNA methylation data reveals the best clustering performance.

## 1. Introduction

DNA methylation is a covalent modification of DNA, which can regulate the expression of genes [1]. Recently, DNA methylation has attracted considerable interest, due its role in the etiology of complex diseases, specially cancer [2], but also because it can be easily measured genome-wide from limited amounts of DNA, allowing measurements in clinical specimens [3].

Relatively little is known about the taxonomy of cancers at the DNA methylation level. Hence, there is currently a strong interest in performing unsupervised clustering of large-scale DNA methylation data sets in order to identify novel cancer subtypes. DNA methylation data is quantified naturally in terms of a beta-distribution. The DNA methylation beta-value, *β*, at a specific genomic locus provides an estimate of the fraction of cells that have that locus methylated. Although studies have considered using the logit-transform *y* = log_2_ *β*/(1 − *β*) instead of *β* for subsequent statistical inferences [4], it was shown in Zhuang *et al.* [5] that the logit-basis can, under certain circumstances, lead to worse inference, as it can aggravate the effects of outliers (*i.e.*, *β* values close to zero or one): from a biological perspective, an outlier at *β* = 0.999 is not more interesting than one at *β* = 0.9, yet on the logit scale, they would be widely separated. Moreover, analyzing DNA methylation data in terms of beta-values helps interpretability. Thus, there is considerable benefit in performing statistical inferences from the original beta-valued distributions. As a result of this, normalization, feature selection and clustering methods designed for beta-valued DNA methylation data have recently been investigated [6,7,8,9,10,11,12,13,14,15].

However, there still remains a significant shortage of methods, specially for the dimensional reduction of large DNA methylation data sets. For instance, blind source separation (BSS) [16,17], independent/principal component analysis (ICA/PCA) [18,19] and nonnegative matrix factorization (NMF) [20,21] techniques have been extensively studied in the gene expression field. Due to the bounded support property of the beta-valued data, the DNA methylation level cannot be efficiently described by these existing dimension reduction methods, which mainly assume the Gaussian distribution of the data. Therefore, the analysis results based on such mismatched models are not sufficiently promising.

Gaussian distribution is the ubiquitous probability distribution used in statistics [22,23,24]. It has an analytically tractable probability density function (pdf), and analysis based on it can be derived in an explicit form. In practice, not all of the data we need to model are Gaussian distributed [25,26]. Recent research showed that, when processing the non-Gaussian distributed data, applying suitable non-Gaussian distribution to model the data can lead to better performance than that obtained by a conventional Gaussian distribution. The advantages of applying a non-Gaussian distribution have been demonstrated in various real-life applications, including image processing [27,28], speech coding [29], document analysis [30], communication and compressive sensing systems [31], complex network analysis [32], decision-making in expert systems [33,34] and biomedical signal processing [35,36]. In particular, non-Gaussian statistical models have also been widely applied in bioinformatics [12,14,15], especially to the analysis of omics data. Indeed, omics data types are rarely Gaussianly distributed.

In this paper, we will introduce and compare some machine learning methods, which are based on non-Gaussian statistical models, for DNA methylation data analysis. The analysis of DNA methylation data includes two parts. (1) Dimensional reduction: DNA methylation array data is high-dimensional, typically involving on the order of 25 k up to 500 k dimensions (and even higher). As with other omics data, the number of samples is typically on the order of 100. However, typically, most of the salient variability in the data, e.g., variation distinguishing cancer from normal samples, or distinguishing different cancer phenotypes, is captured by a much lower-dimensional space. Hence, we need to perform some forms of dimension reduction; (2) Unsupervised clustering: Cancers especially are known to be highly heterogeneous [37]. Hence, we expect an effective and accurate unsupervised learning method to reveal such heterogeneity. Thus, although normal and cancer should be well discriminated, cancer samples may form multiple distinct clusters.

## 2. Results and Discussion

### 2.1. Data Description and Preprocessing

The DNA methylation data is obtained from Gene Expression Omnibus (GEO) website [38]. GEO is a public functional genomics data repository supporting MIAME-compliant data submissions. Dataset GSE32393 [39] is used for the evaluation of the above-mentioned dimension reduction and unsupervised clustering methods. DNA methylation profiles were obtained across approximately 27, 578 CpGs in breast tissues from women with and without breast cancer. Breast tissue samples were drawn from 113 breast cancers and 23 non-neoplastic breast tissues.

**Figure 1:**
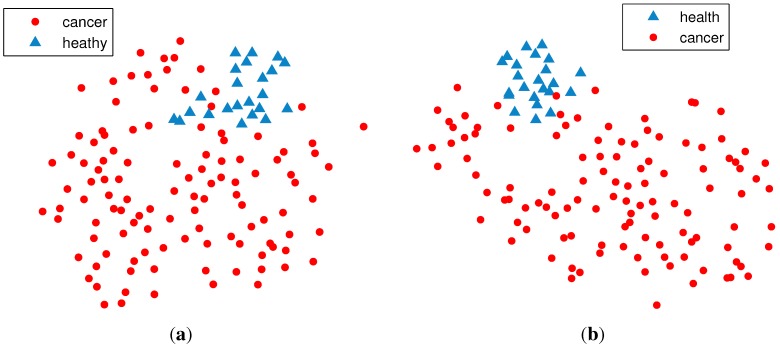
Comparisons of DNA methylation data before and after dimension reduction. (**a**) Visualization of the original 27, 578 dimensional data; (**b**) visualization of the reduced 5000 dimensional data.

We considered a DNA methylation data matrix over 5000 dimensions (specifically, CpG dinucleotides). The 5000 CpGs were selected as those with the highest variance across the 136 samples. A data matrix **X** with a size of 5000 × 136 was obtained. Figure 1 illustrates the effect of dimension reduction. The data points in Figure 1b are separated better than those in Figure 1a, which indicates that dimension reduction can potentially improve the clustering performance. From here and thereafter, the stochastic neighbor embedding (t-SNE) method [40] (the t-SNE method can only approximately illustrate the high-dimensional data for visualization convenience; it is not the exact representation of the relations between data points) is applied to visualize the high-dimensional data in two-dimensional space.

In each dimension reduction method, we need to specify the number of dimensions for which to search. The random matrix theory (RMT) [41] was used for determining the underlying number of dimensions. Although data is distinctly non-Gaussian (even after mean-centering each CpG), RMT provides a reasonable approximation of the dimensionality, as shown by us previously in [16]. For our data matrix of 5000 × 136, we estimated a total of 14 dimensions. Hence, we set *K* = 14 in all of the following experiments.

### 2.2. Unsupervised Clustering

The level of DNA methylation can discriminate normal and cancer samples [42,43]. Cancers especially are also known to be highly heterogeneous [37], which means that the cancer samples should be grouped into more than one cluster. The above obtained matrix **X** still has more features than samples. Therefore, we need to further reduce the dimension and then apply an advanced clustering method to group samples appropriately.

#### 2.2.1. PCA (Principal Component Analysis) + VBGMM (Variational Bayesian Gaussian Mixture Model)

**Figure 2:**
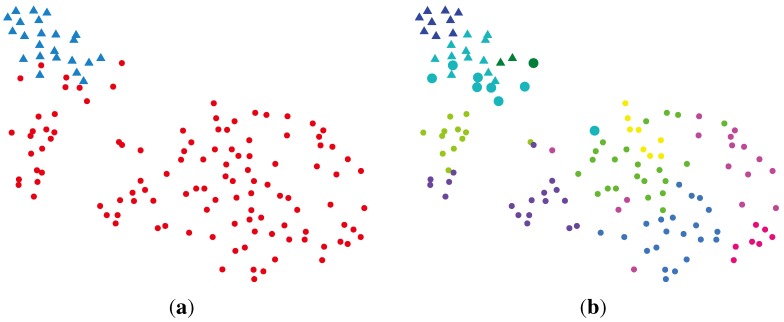
Illustration of the clustering result via PCA (principal component analysis) + the variational Bayesian Gaussian mixture model (VBGMM). The clusters are color coded. The normal data are marked with dots, and the cancer data are marked with crosses. Samples in a larger size are those misclustered. (**a**) Data visualization; (**b**) clustering result via PCA + VBGMM.

When implementing dimension reduction with Gaussian assumptions, PCA [22] is the widely used method. After taking eigenvalue decomposition on the covariance matrix of the observed data, PCA keeps *K* eigenvectors that correspond to the *K* largest eigenvalues. With PCA, a matrix of size 14 × 136 was obtained. For this reduced feature matrix, the variational Bayesian Gaussian mixture model (VBGMM) [22] was applied to estimate the number of clusters, as well as to cluster the data. The VBGMM estimated 10 clusters. Figure 2a illustrates the reduced features’ distribution, and the clustering results are shown in Figure 2b. The PCA + VBGMM misclustered nine samples, out of which nine cancer samples are clustered as normal ones and no normal sample is recognized as a cancer one.

#### 2.2.2. BG-NMF (Beta-Gamma-Nonnegative Matrix Factorization) + RPBMM (Recursive Partitioning Beta Mixture Model)

We applied the BG-NMF method (this work was presented in; it is used as the benchmark for non-Gaussian methods in our paper) to the above mentioned 5000 × 136 matrix. We set the number of basis vectors equal to 14 when applying the BG-NMF method.

Setting the number of basis vectors equal to 14 and applying BG-NMF to **X**^T^ resulted in a 136 × 14 pseudo-basis matrix and a 14 × 5000 excitation matrix. The hypothesis is that the dimensionally-reduced basis matrix, whose element remains bounded, supported and is assumed to be beta distributed, captures the salient patterns of variation.

**Figure 3:**
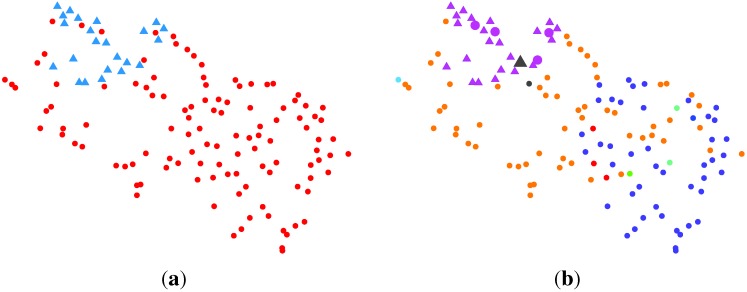
Illustration of the clustering result via beta-gamma (BG)-nonnegative matrix factorization (NMF) + VBBMM (variational Bayesian estimation framework for BMM). The clusters are color coded. The normal data are marked with dots, and the cancer data are marked with crosses. Samples in the larger size are those misclustered. (**a**) Data visualization; (**b**) clustering result via BG-NMF + VBBMM.

The benchmarked RPBMM algorithm was applied to estimate the final clusters of the reduced 136 × 14 matrix, which is illustrated in Figure 3a. The clustering is carried out on a 14-dimensional space. Table 1 lists the final results. RPBMM inferred a total of nine clusters. In summary, five samples (out of 136 samples) were misclustered. Four cancer samples were identified as normal samples, and only one normal sample is identified as cancer. This result is the same as the one obtained by applying RPBMM directly on the original 136 × 5000 data matrix. However, the overall time consumption is reduced from 1275 s to 139 s. Hence, dimension reduction via BG-NMF cannot only yield a convincing clustering result, but also facilitate the calculation.

**Table 1:** Recursive partitioning beta mixture model (RPBMM) clustering details for all of the 136 samples over 14 BG-NMF pseudo-basis vectors.

Cluster	Normal	Cancer
rLLLL	16	4
rLLLR	6	0
rLLR	1	32
rLR	0	31
rRLLL	0	4
rRLLR	0	3
rRLRL	0	10
rRLRR	0	5
rRR	0	34

#### 2.2.3. BG-NMF + VBBMM (Variational Bayesian Estimation Framework for BMM)

One disadvantage of the RPBMM method is that it will estimate the number of mixture components in a recursive manner. It employs the wtdBIC to decide whether to further split one mixture component into two or not. The VBBMM can potentially estimate the model complexity automatically. After convergence, each mixture component corresponds to one cluster. Hence, we applied the VBBMM method on the 136 × 14 pseudo-basis matrix to cluster the samples. The initial settings of the number of mixture component is 15. Eventually, the VBBMM method estimated nine clusters (by removing components whose mixture weights are smaller than 0.01), which is the same as that inferred by RPBMM. Five samples are misclustered, out of which four cancer samples are classified as normal ones, and only one normal sample is recognized as a cancer one. The overall computational time in this scenario is about 124 seconds, which is faster than BG-NMF + RPBMM. This is the main advantage of applying the BG-NMF + RPBMM method. The clustering results are illustrated in Figure 3.

#### 2.2.4. SC (Spectral Clustering) + VBvMM (Variational Inference Framework-based Bayesian Analysis of the vMF Mixture Model)

**Figure 4:**
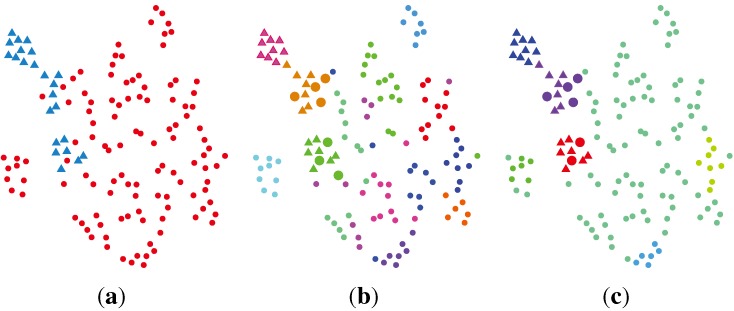
Illustration of the clustering result via spectral clustering (SC) + variational inference framework-based Bayesian analysis of the vMF mixture model (VBvMM) and SC + VBWMM (Watson mixture model (WMM)). The clusters are color coded. The normal data are marked with dots, and the cancer data are marked with crosses. Samples in the larger size are those misclustered. (**a**) Data visualization; (**b**) clustering result via SC + VBvMM; (**c**) clustering result via SC + VBWMM.

An alternative way for dimension reduction is to apply the SC method. As the RMT estimated *K* = 14, we set the target dimension *K* in the SC method to be 14. With the method described in Algorithm 1, a feature matrix of a size of 14 × 136 was obtained. This matrix is visualized in Figure 4a. The parameter *σ* was empirically set as the squared root of the data’s variance in matrix **B**, where *B_ij_* = ||**x***_i_* − **x***_j_*||^2^. This choice is due to the fact that the affinity matrix **A** in Algorithm 1 takes the form of a Gaussian kernel function. Therefore, it is natural to consider the variance of data as *σ*^2^. The choice of the optimal *σ* in the SC method is an open topic for future research. The reduced feature from SC has a unit length, which means the *l*_2_ norm of each feature equals one. To model such property efficiently, the VBvMM is used to realize the unsupervised clustering. After convergence, VBvMM determined 14 clusters. Seven normal samples are clustered as cancer ones, while zero cancer samples are clustered as normal ones. The overall number of misclustered samples is seven. Figure 4b illustrates the clustering result.

#### 2.2.5. SC + VBWMM (Variational Bayesian Estimation of WMM)

Assuming what we observed from the SC resulting feature is only one of the mirrored pairs (with respect to the origin), the VBWMM can be applied to model the axially symmetric data. With a similar approach as the SC + VBvMM, we have six cancer samples clustered as normal ones and none of the normal samples inferred as the cancer one. The overall misclustered samples are six. The VBWMM inferred seven clusters in the end. The clustering results are shown in Figure 4c.

#### 2.2.6. Discussion

The comparisons of the above-mentioned four methods are listed in Table 2. All of the above-mentioned algorithms can yield appropriate unsupervised clustering results. In general, the non-Gaussian distribution-based methods are superior to the Gaussian distribution-based method. This is due to the fact that the Gaussian distribution cannot describe the bounded/unit length property of the features properly. Among all four non-Gaussian distribution-based methods, the BG-NMF + RPBMM/BG-NMF + VBBMM methods outperform the SC + VBvMM/SC + VBWMM methods. This is mainly due to the fact that bounded support is an important property of DNA methylation data [5]. The BG-NMF methods yield reduced features that retain the bounded property of the data, while the SC-related methods produced a feature (which has a unit length) that does not directly reflect the bounded support property. The BG-NMF + VBBMM performs the best among all three proposed methods. It also outperforms the benchmarked BG-NMF + RPBMM method in terms of computational cost, which is because of the advantage that the variational Bayesian method can automatically determine the complexity.

**Table 2:** Comparisons of the clustering performance of different methods.

Method	Error Rate	Cancer→Normal	Normal→Cancer
**PCA + VBGMM**	6.62%	9	0
**BGNMF + RPBMM**	3.68%	4	1
**BGNMF + VBBMM**	3.68%	4	1
**SC + VBvMM**	5.15%	7	0
**SC + VBWMM**	4.41%	6	0

When looking at the misclustered samples, all of the BG-NMF related clustering methods miscluster four or five cancer samples to normal and miscluster one normal sample to cancer, while the SC-related method estimated six or seven cancer samples to normal, but no normal sample to cancer. Misclustering happens since the data are highly-dimensionally correlated. Although we have reduced the dimensions to remove redundant features, it is still difficult to separate one type of data from the other. The SC-related methods, however, do not miscluster any normal sample to cancer. We speculate that this is because the SC method embedded the data in a tight manner, so that a relatively “clearer” positive/negative boundary can be obtained than the BG-NMF method. On the one hand, BGNMF-related methods have overall better clustering performance than the SC-related methods, but misclustered data in both ways. On the other hand, SC-related methods do not cluster any normal data to cancer, but have relatively worse overall accuracy. These observations motivate us to improve the unsupervised clustering method so that better clustering results can be obtained.

In summary, for DNA methylation analysis, the bounded nature of the data plays an important role. Thus, such a property should be retained in both the dimension reduction and clustering methods. Furthermore, an appropriate unsupervised learning method is required for revealing the heterogeneity more accurately.

## 3. Experimental Section

### 3.1. Non-Gaussian Statistical Distributions

The Gaussian distribution (both univariate and multivariate) has a symmetrical “bell” shape, and the variable’s definition is on the interval (−∞, ∞). Non-Gaussian statistical distributions refer to a set of distributions that have special properties that the Gaussian distribution cannot characterize. For example, the beta distribution is defined on the interval [0, 1] (in a general form, the beta distribution could have definition on any interval [a,b]; after linear scaling, it can be represented with the standard beta distribution [44]) and can have a symmetric or asymmetric shape [27]. The Dirichlet distribution, which is a multivariate generalization of the beta distribution, has a pdf with respect to the Lebesgue measure on the Euclidean space [45]. The gamma distribution is defined on the interval (0, ∞), and the shape cannot be symmetric [46]. To model data whose *l*_2_ norm equals one, the von Mises–Fisher (vMF) distribution [47] and the Watson distribution [48] are usually applied. These distributions show characteristics that are significantly different from a Gaussian distribution.

In the remaining part of this section, we will introduce some typical non-Gaussian distributions that can be applied in DNA methylation analysis.

#### 3.1.1. Beta Distribution

**Figure 5:**
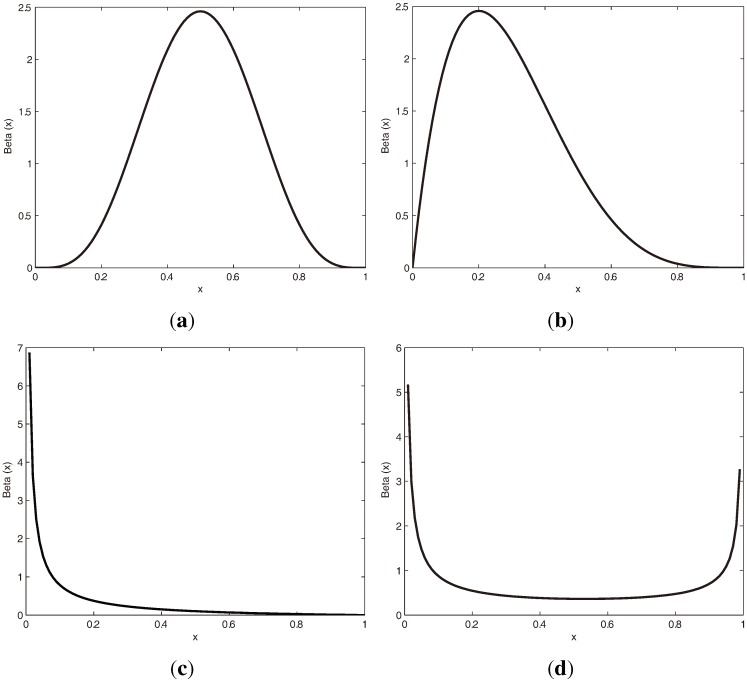
Beta distributions for different pairs of parameters. (**a**) *u* = 5, *v* = 5; (**b**) *u* = 2, *v* = 5; (**c**) *u* = 0.1, *v* = 2; (**d**) *u* = 0.2, *v* = 0.8.

The beta distribution is characterized by two positive shape parameters *u* and *v*. The pdf of the beta distribution is:

(1) $$\text{Beta}(x;u,v)=\frac{\Gamma (u+v)}{\Gamma (u)\Gamma (v)}x^{u-1}{\left(1-x\right)}^{v-1}$$

where Γ(·) is the gamma function. The beta distribution has a flexible shape, which is shown in Figure 5. In real applications, the beta distribution can be applied to model the distribution of a gray image pixel [49], to describe the probability of human immunodeficiency virus (HIV) transmission [50] and to capture the bounded property of the DNA methylation level [8,15].

#### 3.1.2. vMF (von Mises-Fisher) Distribution

**Figure 6:**
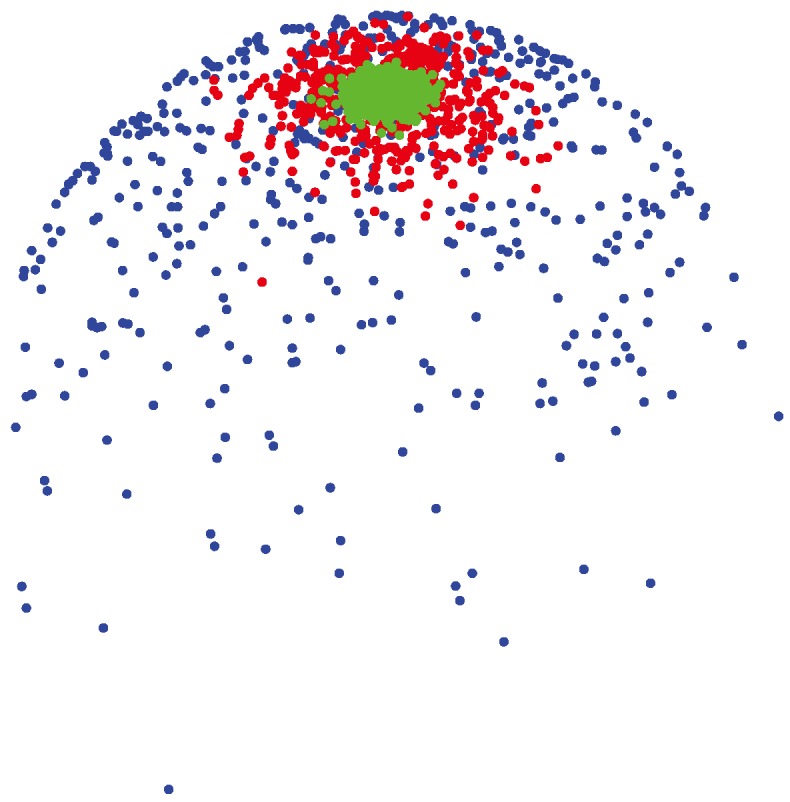
Scatter plot of samples from a single von Mises–Fisher (vMF) distribution on the sphere for different concentration parameters, *λ* = {4, 40, 400}, and around the same mean direction ***μ*** = [0, 0, 1]*^T^*. Samples generated from 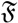 (***μ***, 400) (shown by green colors) are highly concentrated around the mean direction, while for samples generated from 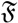 (***μ***, 4) (shown by blue colors), the distribution of samples on the sphere is more uniform around the mean direction.

The vMF distribution is considered a popular distribution in the family of directional distributions [47,51]. The data following a vMF distribution are located on a unit hypersphere. Hence, the vMF variable’s *l*_2_ norm equals one, *i.e.*, ||**x**||_2_ = 1. The vMF distribution contains two parameters, namely the mean direction ***μ*** and the concentration parameter *λ*. The pdf of a *K*-dimensional vMF distribution can be expressed as:

(2) F( x∣ μ,λ)=cK(λ) eλ μT x

where ||***μ***||_2_ = 1, *λ* ≥ 0 and *K* ≥ 2 [51]. The normalizing constant c*_K_*(*λ*) is given by:

(3) $$c_{K}(\lambda )=\frac{\lambda^{\frac{K-2}{2}}}{{\left(2\pi \right)}^{\frac{K}{2}}I_{\frac{K-2}{2}}(\lambda )}$$

where ℐ*_ν_*(·) represents the modified Bessel function of the first kind of order *ν* [52]. The pdf of the vMF distribution is illustrated in Figure 6. In information retrieval applications, the vMF distribution can be applied to model the cosine similarity for the clustering of text documents [53,54]. It can also be applied in modeling the gene expression data, which has been shown to have directional characteristics [55].

#### 3.1.3. Watson Distribution

Observations on the sphere might have an additional structure, such that the unit vectors **x** and −**x** are equivalent. In other words, that is ±**x** that are observed. Here, we need probability density functions for **x** on 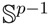 which are axially symmetric, that is *f*(−**x**) = *f*(**x**). In such cases, the *p*-dimensional observation ±**x** can be regarded as being on the projective space ℙ*^p^*^−1^, which is obtained by identifying opposite points on the sphere 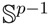.

One of the simplest distributions for axial data, with a rotational symmetry property, is the (Dimroth–Scheidegger–) Watson distribution. The Watson distribution is a special case of the Bingham distribution [56], which is developed for axial data with no rotational symmetry property.

**Figure 7:**
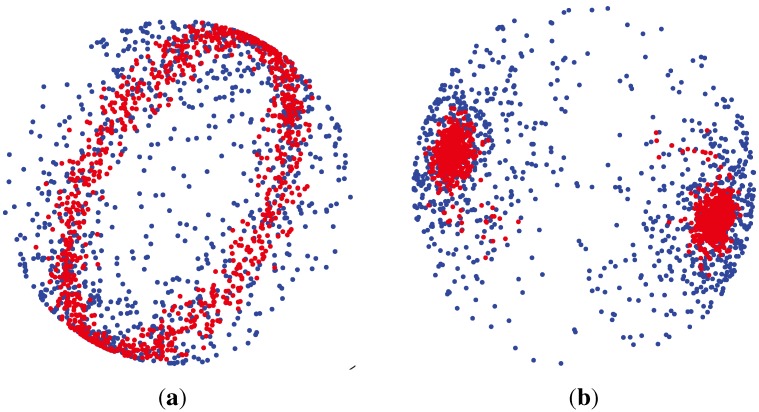
Scatter plot of samples from a single distribution, *W_p_* (***μ***, *κ*), on the sphere for positive and negative concentration parameters *κ*, around the same mean direction, *μ* = [0, 1]^T^ [60]. For larger concentration parameters, *i.e*., *κ* = 40 or *κ* = −40, samples are more concentrated around the mean direction (shown by the red color). For smaller concentration parameters, *i.e.*, *κ* = 4 or *κ* = −4, samples are more uniformly distributed around the mean direction (shown by the blue color). (**a**) *κ* > 0, *κ* ∈ {+4, +40}; (**b**) *κ* < 0, *κ* ∈ {4, −40}.

A random vector **x** ∈ ℙ*^p^*^−1^, or equivalently ±**x** ∈ 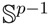, has the (*p* − 1)-dimensional Watson distribution *W_p_* (***μ***, *κ*), with the mean direction ***μ*** and the concentration parameter *κ*, if its probability density function is:

(4) Wp( x∣ μ,κ)=Γ(p)(2π)pF11(r,p,κ)(κ)eκ‖ μ† x‖2

where *κ* ∈ ℝ, ||***μ***||_2_ = 1, and _1_*F*_1_ is Kummer’s (confluent hypergeometric) function (e.g., [57] (Formula (2.1.2)), or [58] (Chapter 13)), defined as:

(5) F11(r,p,κ)=∑j≥0r(j)p(j)κjj!

where $r^{\left(j\right)}\equiv \frac{\Gamma (r+j)}{\Gamma (r)}$ is the raising factorial. Similar to the case of vMF distributions, for *κ* > 0, as *κ* → 0, *W_p_* (***μ***, *κ*) reduces to the uniform density, and as *κ* → ∞, *W_p_* (***μ***, *κ*) tends to a point density. For *κ* < 0, as *κ* → − ∞, the density concentrates around the great circle orthogonal to the mean direction ([59] (Chapter 9.4)). The samples generated from Watson distribution are shown in Figure 7.

### 3.2. Non-Gaussian Dimension Reduction Methods

When analyzing DNA methylation data, the high-dimensional property presents mathematical challenges, as well as opportunities. The main purpose of applying dimension reduction methods on microarray data is to extract the core features driving interesting biological variability [61]. Such methods include principal component analysis (PCA) [22,62], nonnegative matrix factorization (NMF) [5,63] and singular value decomposition (SVD) [64].

### 3.3. Nonnegative Matrix Factorization for Bounded Support Data

Unlike PCA or ICA, NMF reveals the data’s nonnegativity during dimension reduction. Traditional NMF decomposes the data matrix into a product of two nonnegative matrices as:

(6) XP×T≈ WP×K VK×T

where **X***_P×T_*, **W***_P×K_* and **V***_K×T_* contain nonnegative values *X_pt_*, *W_pk_* and *V_kt_*, respectively, and *p* = 1, …, *P*, *t* = 1, …, *T*, *k* = 1, …, *K*, *K* ≪ *T*.

The DNA methylation data are naturally bounded on interval [0, 1]. Conventional NMF strategies do not take such a nature into account. In order to capture such a bounded feature explicitly, we proposed an NMF for bounded support data [65]. Each bounded support element *X_pt_* is assumed to be generated from a beta distribution with parameters *a_pt_* and *b_pt_*. With an observation matrix **X***_P×T_*, two parameter matrices **a** and **b** of size *P* × *T* are obtained, respectively. Each parameter matrix, rather than the observation matrix, is decomposed into a product of a basis matrix and an excitation matrix as:

(7) aP×T≈ AP×K HK×T bP×T≈ BP×K HK×T

With the above description, we assume that the matrix **X** (with element *X_pt_* ∈ [0, 1]) is drawn according to the following generative model:

(8) $$\begin{array}{l} A_{pk}\sim \text{Gamma}(A_{pk};\mu_{0},\alpha_{0})\\ B_{pk}\sim \text{Gamma}(B_{pk};\nu_{0},\beta_{0})\\ H_{kt}\sim \text{Gamma}(H_{kt};\rho_{0},\zeta_{0})\\ X_{pt}\sim \text{Beta}(X_{pt};\sum_{k}A_{pk}H_{kt},\sum_{k}B_{pk}H_{kt}) \end{array}$$

where Gamma(*x; k*, *θ*) is the gamma density with parameters *k*, *θ* defined as:

(9) $$\text{Gamma}(x;k,\theta )=\frac{\theta^{k}}{\Gamma (k)}x^{k-1}e^{-\theta x},k,\theta >0$$

As the data is assumed to be beta distributed and the parameters of the beta distribution are assumed to be gamma distributed, this model is named BG-NMF.

For BG-NMF, the variational inference (VI) method [66] is applied to estimate the posterior distributions. The expected value of *X_pt_* is ${\bar{X}}_{pt}=\frac{a_{pt}}{a_{pt}+b_{pt}}$. If we take the point estimate to *A_pk_*, *B_pk_* and *H_kt_*, then the expected value of *X_pt_* can be approximated as:

(10) $${\bar{X}}_{pt}\approx \frac{\sum_{k}{\bar{A}}_{pk}{\bar{H}}_{kt}}{\sum_{k}{\bar{A}}_{pk}{\bar{H}}_{kt}+\sum_{k}{\bar{B}}_{pk}{\bar{H}}_{kt}}$$

which can be expressed in matrix form as:

(11) $$\bar{X}\approx (\bar{A}\;\bar{H})\emptyset (\bar{A}\;\bar{H}+\bar{B}\;\bar{H})$$

where ⊘ means element-wise division. When placing sparsity constraints on the columns in **H**, the reconstruction in Equation (11) could be approximated as:

(12) $$\bar{X}\approx [\bar{A}\emptyset (\bar{A}+\bar{B})]\;\bar{H}=\bar{W}\;\bar{H}$$

Hence, the resulting pseudo-basis matrix **W̄** is low-dimensional while retaining the bounded support constraint.

#### 3.3.1. Spectral Clustering for Non-Gaussian Reduced Features

Recently, spectral clustering (SC) has become one of the most popular clustering algorithms [67]. It is an alternative method for the K-means algorithm. When the natural clusters in ℝ*^L^* are not corresponding to the convex region, the K-means algorithm cannot provide satisfactory clustering results. However, when mapping the data points to ℝ*^K^* space via SC, they may form tight clusters [68]. SC analyzes the affinity matrix of data. Assuming that the data are likely to be clustered in *K*-dimensional space, the reduced features, each of which is *K*-dimensional, are extracted by taking eigenvalue analysis of an intermediate matrix **M**. The reduced features will then be used for clustering, with conventional methods like K-means. The feature extraction procedure via the SC method [68] is summarized in Algorithm 1. With the above extracted features, the task of data clustering can be carried out in the reduced ℝ*^K^* space.

**Algorithm 1:** Spectral clustering.

**Input:** Original data matrix **X** = {**x**_1_, **x**_2_, …, **x***_N_*}, each column is a *L*-dimensional vector. Create the affinity matrix **A***_N_*_×_*_N_*, where $A_{ij}=\{\begin{array}{cc} e^{\frac{-{\left\|x_{\text{i}}-x_{\text{j}}\right\|}^{2}}{2\sigma^{2}}} & i\neq j\\ 0 & i=j \end{array}$; Construct the intermediate matrix M= D-12 AD-12, where **D** is a diagonal matrix whose (*i*, *i*)th element is the summation of the *i*-th row of **A**; Apply eigenvalue analysis on **M** and create a matrix **Y***_K_*_×_*_N_*, which contains *K* eigenvectors corresponding to the largest *K* eigenvalues; Form a matrix **Z** from **Y** by normalizing each column of **Y**. Each column of **Z** has a unit length. **Output:** Reduced *K*-dimensional feature **z***_n_* for each data point **x***_n_*.

### 3.4. Non-Gaussian Statistical Models for Unsupervised Clustering

The ultimate goal of dimension reduction is to benefit the clustering of DNA methylation data. The dimension reduction methods introduced above yield features with special properties. The BG-NMF method provides a basis matrix (see Equation (12)), every element of which is on the interval [0, 1]. The SC method constructs feature vectors with a unit length in the reduced *K*-dimensional space. These data are obviously non-Gaussian distributed. It has been shown in several studies that applying a suitable non-Gaussian distribution to model the non-Gaussian distributed data can improve the corresponding performance [25,26,31]. To this end, we apply the non-Gaussian distribution that can nicely describe the data distribution to realize the clustering task in the reduced feature space.

#### 3.4.1. Recursive Partitioning Beta Mixture Model

For the beta-valued DNA methylation data, it is natural to consider the beta distribution as a candidate to model the underlying distribution. Since the DNA methylation data that show an obvious normal/cancer status are multi-modal, a beta mixture model (BMM) can be applied for modeling. One mixture component represents one cluster. In unsupervised clustering, selecting the optimal number of clusters is a big challenge. One popular method designed for such purpose is the recursive partitioning beta mixture model (RPBMM) [12]. The RPBMM navigates clusters in a BMM. It treats each mixture component as a node and takes the weighted version of Bayesian information criterion (wtdBIC) to decide whether to stop further recursive partitioning of the current node. In the end, the optimal number of clusters is determined. This is a recursive unsupervised learning method. In this paper, the clustering results based on the RPBMM method are taken as the benchmark.

#### 3.4.2. Variational Beta Mixture Model

Another way of carrying out model selection is to employ the variational Bayesian estimation framework for BMM (VBBMM). Under this circumstance, the joint posterior distribution of the weighting factors is modeled by a sparse Dirichlet distribution, so that the component with a very small weight will be pruned from the mixture model. In [27], the extended variational inference strategy is applied to derive an analytically tractable solution for estimating the parameters in a BMM. The proposed algorithm can remove the redundant component. This closed-form solution avoids the cross-validation in the methods using BIC as the criterion (e.g., [12,44]). Hence, it is computationally efficient, and the unsupervised clustering is facilitated.

#### 3.4.3. Variational von Mises-Fisher Mixture Model

The data with its *l*_2_ norm equaling one has a directional property. The von Mises-Fisher (vMF) distribution is suitable for such a type of data [47,69]. In order to decide the model complexity (in terms of free parameters) automatically based on the data, we proposed a variational inference framework-based Bayesian analysis of the vMF mixture model (VBvMM) in [47]. This method can potentially determine the model complexity and avoid the over-fitting problem associated with conventional approaches based on the expectation maximization. This variational vMM is a suitable model for the unsupervised clustering of directional data.

#### 3.4.4. Variational Watson Mixture Model

The Watson distribution is a simple distribution for modeling axially symmetric data on the unit hypersphere ([59] (Chapter 9.4)). By assuming that any data point has its axial mirror, it is natural to model the distribution of the data with the unit length (*i.e.*, *l*_2_ norm equals one) and its axial mirror by the Watson distribution. Similarly, when such data are multi-modally distributed, a Watson mixture model (WMM) can be applied. With a variational inference framework, Taghia *et al.* [60] proposed the variational Bayesian estimation of WMM (VBWMM), where the model complexity can be determined by pruning the irrelevant components. This variational WMM can also be applied for the purpose of unsupervised clustering.

## 4. Conclusions

Cancer is characterized by alterations at the DNA methylation level. A Gaussian distribution, in general, cannot describe the DNA methylation data appropriately. Hence, the Gaussian distribution-based unsupervised clustering does not provide convincing performance.

For the purpose of efficiently clustering DNA methylation data, we proposed several dimension reduction methods and consequent unsupervised learning methods, which are all based on non-Gaussian distributions. They all perform better than the Gaussian distribution-based method. In the dimension reduction step, both the BG-NMF and the SC methods can remove the redundant dimensions efficiently. In unsupervised clustering, the VBBMM method, the VBvMM method and the VBWMM method can all reveal the heterogeneity of the DNA methylation data appropriately. Clustering performance demonstrates that the proposed non-Gaussian distribution-based methods are meaningful tools for analyzing DNA methylation data. Experimental results also show that the BG-NMF + VBBMM method performs the best among all of the proposed methods and is faster than the benchmarked BG-NMF + RPBMM method. Furthermore, for the reduced features inferred from both the BG-NMF method and the SC method, the consequent unsupervised clustering method needs to be improved, so that better clustering accuracy can be obtained.

Moreover, the methodology introduced in this paper can be easily extended to analyze other DNA methylation data sets. Some other non-Gaussian statistical models can also be applied for such purposes.
